# Reinforcement of Hydrogels with a 3D-Printed Polycaprolactone (PCL) Structure Enhances Cell Numbers and Cartilage ECM Production under Compression

**DOI:** 10.3390/jfb14060313

**Published:** 2023-06-07

**Authors:** Hamed Alizadeh Sardroud, Xiongbiao Chen, B. Frank Eames

**Affiliations:** 1 Division of Biomedical Engineering, College of Engineering, University of Saskatchewan, Saskatoon, SK S7N 5A9, Canada; xbc719@mail.usask.ca; 2 Department of Mechanical Engineering, College of Engineering, University of Saskatchewan, Saskatoon, SK S7N 5A9, Canada; 3 Department of Anatomy, Physiology, and Pharmacology, University of Saskatchewan, Saskatoon, SK S7N 5E5, Canada

**Keywords:** compressive force, hydrogel, reinforced, unreinforced, cell numbers, cartilage ECM, hyaline cartilage, fibrocartilage, Col1, Col2

## Abstract

Hydrogels show promise in cartilage tissue engineering (CTE) by supporting chondrocytes and maintaining their phenotype and extracellular matrix (ECM) production. Under prolonged mechanical forces, however, hydrogels can be structurally unstable, leading to cell and ECM loss. Furthermore, long periods of mechanical loading might alter the production of cartilage ECM molecules, including glycosaminoglycans (GAGs) and collagen type 2 (Col2), specifically with the negative effect of stimulating fibrocartilage, typified by collagen type 1 (Col1) secretion. Reinforcing hydrogels with 3D-printed Polycaprolactone (PCL) structures offer a solution to enhance the structural integrity and mechanical response of impregnated chondrocytes. This study aimed to assess the impact of compression duration and PCL reinforcement on the performance of chondrocytes impregnated with hydrogel. Results showed that shorter loading periods did not significantly affect cell numbers and ECM production in 3D-bioprinted hydrogels, but longer periods tended to reduce cell numbers and ECM compared to unloaded conditions. PCL reinforcement enhanced cell numbers under mechanical compression compared to unreinforced hydrogels. However, the reinforced constructs seemed to produce more fibrocartilage-like, Col1-positive ECM. These findings suggest that reinforced hydrogel constructs hold potential for in vivo cartilage regeneration and defect treatment by retaining higher cell numbers and ECM content. To further enhance hyaline cartilage ECM formation, future studies should focus on adjusting the mechanical properties of reinforced constructs and exploring mechanotransduction pathways.

## 1. Introduction

Hydrogels are polymeric networks containing 60–90% water that stand out for use in cartilage tissue engineering (CTE) [1,2,3]. The high water content of hydrogels simulates highly hydrated articular cartilage in humans and provides chondrocytes with a three-dimensional (3D) environment to maintain their morphology and phenotype [4,5,6]. Moreover, hydrogels are biocompatible and biodegradable, which makes them suitable candidates for cartilage construction development as well [6]. It appears that hydrogels like alginate [7], agarose [8], and collagen [9] offer the best performance among various existing biomaterials. As a cheap biomaterial, alginate is advantageous over other hydrogels since it can be cross-linked at room temperature without organic solvents and can be used to prepare a variety of shapes [10]. Alginate hydrogels impregnated with chondrocytes have shown promise as engineered constructs for articular cartilage repair by producing cartilage markers like glycosaminoglycans (GAGs) and collagen type 2 (Col2) [11,12,13], which are the main components of the articular hyaline cartilage in joints [14].

Several fabrication techniques have been successfully used to impregnate cells within hydrogels [5,15,16], and among them, 3D-bioprinting has proven to be promising for the fabrication and transplanting of different kinds of tissues, including skin, bone, cartilage, the heart, vascular grafts, and other vital organs [17,18,19,20,21]. Definable dimensions and architectural characteristics of fully interconnected 3D hydrogel structures can be formed by dispensing a layer-by-layer solution (bioink) onto a platform through 3D-bioprinting [22,23,24]. 3D-bioprinted hydrogels may provide significant benefits for CTE [25,26], but their poor mechanical properties and low 3D structural integrity are major drawbacks for long-period mechanical compressions in vitro and as required in vivo implantations [27,28]. Hydrogels may not offer sufficient 3D structural integrity during surgical procedures and post-surgery regeneration due to their relatively low to moderate mechanical properties [29].

Researchers are therefore exploring synthetic polymers with strong mechanical and integrity properties for load-bearing applications [30]. By using synthetic polymers in 3D printing, scaffolds with durable stability can be fabricated to withstand the force or loading on the joints [31]. Polycaprolactone (PCL), which gained approval from the US Food and Drug Administration (FDA) in 2006, is a biodegradable synthetic biomaterial with excellent mechanical properties that has been extensively used in CTE applications [32,33]. Synthetic polymers, however, do not provide an adequate environment for cells due to their hydrophobic properties [34]. Thus, it is rational to develop scaffolds made from hydrogels while being reinforced with synthetic polymer(s) to provide a supportive structure [33,35]. It has been demonstrated that such hydrogel-reinforced constructs can regenerate cartilage matrix in vitro while maintaining mechanical and structural integrity [36,37], yet the mechanical properties of a hydrogel construct can affect cell growth as well as chondrogenic activities through cell–ECM interactions and mechanical cues from cells surrounding the hydrogel [38,39]. While deposition of collagen type 2 (Col2) is crucial for hyaline cartilage formation, increased deposition of collagen type 1 (Col1) leads to fibrocartilage formation instead [14,40,41]. Previous studies mostly assessed Col2 and GAG production within hydrogel constructs subjected to mechanical forces without considering Col1. However, due to the fact that fibrocartilage forms with traditional treatments including morrow stimulation and microfractures [42,43,44] and the existence of a few studies that reported Col1 production in mechanically loaded hydrogels in vitro and in vivo [45,46,47], it is likely that cells produce Col1 when they undergo mechanical forces. Thus, it is urged that both Col1 and Col2 deposition need to be assessed within constructs subjected to mechanical forces [48].

This study aimed to investigate the impact of extending the compression period on hydrogel performance in terms of cell numbers and ECM production, specifically focusing on the differentiation between hyaline-like cartilage and fibrocartilage-like ECM production. Initially, unreinforced hydrogel constructs, which are softer structures with large pore sizes, were examined. These constructs have the potential to lose their 3D integrity under compression, leading to cell and ECM release. In addition, reinforced hydrogel constructs were fabricated by incorporating 3D-printed PCL scaffolds into a cell-alginate mixture. The aim was to assess whether the reinforcement would enhance performance compared to unreinforced hydrogels. It is important to note that this study focused solely on compression as one of the predominant forces acting within the knee joint [49,50].

## 2. Materials and Methods

### 2.1. Cell Culture

The murine cell line ATDC5 is recognized for its ability to undergo chondrogenesis. Therefore, due to its well-established nature as an in vitro model for the chondrogenic process, the ATDC5 cell line was chosen for utilization in this study [51,52]. The frozen cells obtained from the RIKEN cell bank (Japan) were thawed and placed in a petri dish. They were then cultured in complete media consisting of Dulbecco’s modified Eagle’s medium/Ham’s nutrient mixture F12 (DMEM/F12, Sigma, D8900, USA) supplemented with 5% (*v*/*v*) fetal bovine serum (FBS, Gibco, USA) and 1% (*v*/*v*) penicillin/streptomycin (Sigma, USA). The cells were incubated at 37 °C and 5% CO_2_, with the media being changed every other day. The cells at confluence were treated with 0.25% (*v*/*v*) trypsin (Hyclone, USA) to detach them and then centrifuged at 1000 rpm for 5 min. The cell pellets were collected and cultured in a 175 T-flask with complete media. This process was repeated iteratively until the desired cell numbers were achieved. Once the constructs (reinforced and unreinforced) were fabricated, they were transferred into 6-well plates containing differentiation media. The differentiation media consisted of complete media supplemented with 1% insulin-transferrin-selenium (ITS, Sigma, I3146) and 1% ascorbate-2-phosphate (Sigma, A8960). This step was intended to initiate chondrogenic differentiation. The constructs were cultured in the differentiation media for 10 days, with media changes every alternate day.

It is important to note that during the entire duration of the loading experiments, both the loaded and unloaded constructs were cultured in differentiation media to maintain the chondrogenic differentiation of the cells within the 6-well plates. However, during the compression periods when the loading experiments were conducted (3 h/day), a regular DMEM-F12 medium without additional supplements was used.

By following this protocol, the ATDC5 cells were induced to differentiate into chondrocytes, and the subsequent experiments were carried out accordingly.

### 2.2. Fabrication of Reinforced and Unreinforced Constructs

A 3.6% *w*/*v* alginate solution was prepared by dissolving medium-viscosity alginate (Sigma-Aldrich, A2033) in Stemline Keratinocyte Medium II-Calcium free (Sigma-Aldrich, S0196) under sterile conditions. Trypsinized ATDC5 cells were mixed with alginate solution at a ratio of 3:7, resulting in a cell-alginate mixture with a final cell density and alginate concentration of 5 × 10^6^ cells/mL and 2.5% *w*/*v*, respectively. The resulting cell-alginate mixture was used to fabricate reinforced and unreinforced constructs.

*Fabrication of reinforced constructs:* Using a 3D-bioplotter machine (Envisiontec, Gladbeck, Germany), PCL beads (Sigma-Aldrich, Mw 48,000–90,000) were loaded in a high-temperature dispensing head, heated, and maintained at 65–80 °C for 15–20 min before dispensing. For PCL structure fabrication, 0.8 MPa pressure was used to dispense PCL through a metal needle with an inner diameter of 300 µm (24 G). Five layers of PCL strands, at about 2 mm in height, with 0°/90° strand orientation, 1 mm interstrand spacing, and an 8 mm diameter circular shape, were dispensed using the 3D-bioplotter machine to fabricate PCL scaffolds. Fabricated PCL scaffolds were immersed in a cell-alginate solution and dipped several times to allow the mixture to diffuse into the pores of the PCL scaffolds. Afterward, PCL-alginate constructs were transferred into a 6-well plate containing 50 Mm calcium chloride (CaCl_2_, Sigma-Aldrich). The constructs were kept inside the calcium chloride (CaCl_2_, Sigma-Aldrich) solution for 20 min to crosslink the alginate solution. The reinforced constructs were then rinsed twice with DMEM-F12 media inside a 6-well plate to remove excess CaCl_2_.

*Fabrication of unreinforced hydrogel constructs:* These constructs were fabricated using a 3D-bioprinting method utilizing the 3D-bioplotter machine. A polyethyleneimine (PEI, Alfa Aesar, Mw: 60,000, USA) solution of 0.1% (*w*/*v*) was used to coat sterile 6-well plates overnight at 37 °C. The next day, the PEI solution was removed from the wells, followed by two washes with PBS and one wash with DMEM-F12 media. The 6-well plate was then filled with sterilized 50 Mm CaCl_2_ plus a 0.1% PEI solution and placed on the printing stage. Using the 3D-bioplotter machine, the cell-alginate bioink was plotted in a layer-by-layer manner into the crosslinking solution with 0°/90° strand orientation and 1 mm interstrand spacing to fabricate hydrogel constructs. Several cylindrical hydrogel constructs were fabricated, featuring a diameter of 8 mm and an approximate height of 1.5 mm. Subsequently, the unreinforced constructs were moved to a 6-well plate containing DMEM-F12 media and underwent two rinses with the media to effectively remove any excess CaCl_2_.

After finishing the fabrication of reinforced and unreinforced constructs, they were transferred into the 6-well plates containing differentiation media and cultured for 10 days until starting the compression experiments.

### 2.3. Dynamic Culture of Constructs

Dynamic compression experiments were conducted on both the reinforced and unreinforced constructs using a bioreactor (ElectroForce BioDynamic 5200, TA Instruments, Eden Prairie, MN, USA), as described in our previous study [28]. In brief, four constructs, either reinforced or unreinforced, were placed on a platen inside a chamber of the bioreactor. The upper platen was fixed while the lower platen was being moved up and down to apply a compressive force. DMEM-F12 media was circulated into the chamber using a peristaltic pump while the reservoir of the media was maintained inside a 37 °C water bath during compression. A dynamic sinusoidal compressive force regime with 12% strain, which resembles normal joint activities [53], at 1 Hz frequency for 3 h/day was applied to the constructs. Our experimental setup employed an unconfined compression culture to mimic in vivo compressive forces in a simplified manner. While not replicating the complete mechanical complexity of confined in vivo conditions, this approach allowed us to investigate the biological response of cells within the constructs under dynamic compression as one of the major force types in the knee joint [49,50].

**Figure 1:**
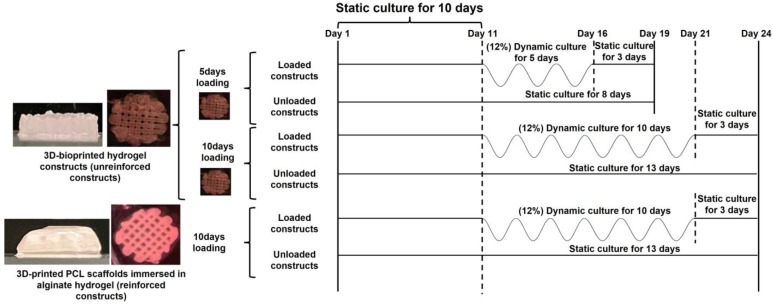
Schematic of the experimental conditions used in this study to compress unreinforced and reinforced constructs. In the figure, sinusoidal waveforms depict dynamic compression inside the bioreactor with 12% strain at 1 Hz frequency for 3 h/day, and horizontal lines indicate a static culture of the constructs in 6-well plates.

To observe how extending the compression period affected cell numbers and ECM production in the unreinforced hydrogel constructs, compressions were performed for 5 and 10 days. Due to the superior mechanical properties of the PCL structure, reinforced constructs had a higher chance of retaining the hydrogel component, and therefore, they were dynamically cultured only for a longer period (10 days of compression, referred to hereafter as reinforced-10 days). Cell numbers and ECM production in the reinforced-10 days constructs were compared with the unreinforced constructs cultured for 10 days (referred to hereafter as unreinforced-10 days). Every loading group of constructs also had a control, unloaded group that was cultured in static conditions in 6-well plates for the duration of the experiments. Upon finishing the compression experiments, the constructs were placed in the 6-well plates containing differentiation media for a period of three days. This timeframe allowed the cells sufficient time to respond to the applied forces. A schematic of experimental conditions is also depicted in Figure 1. 

### 2.4. Post-Culture Analyses

Constructs cultured in static and dynamic conditions were cut in half after their static and dynamic culture periods were over. Half of each construct was fixed and embedded in an optimal cutting temperature medium (OCT, Tissue-Tek) as described in our previous study [28] to use for histology and immunofluorescence staining analyses. The remaining halves of the constructs were further divided into halves, resulting in two-quarters of each construct. These two-quarters were then weighed using a scale and stored in microtubes at −80 °C for subsequent biochemical analysis.

#### 2.4.1. Histological Analysis

A cryotome (Fischer Scientific, USA) was used to section the OCT blocks of the constructs to a thickness of 10 µm. The sections were stored in a −20 °C freezer for histological and immunofluorescence staining to assess cartilage ECM deposition.

A histological analysis of Alcian blue staining was employed on the sections to detect GAG depositions. The sections were subjected to staining with a 0.25% Alcian blue solution in a 3% acetic acid solution for a duration of 4 h. Subsequently, the sections underwent destaining by using graded ethanol solutions in 3% acetic acid over a period of 3 h. The destaining process involved successive ethanol concentrations of 50%, 75%, and 100%. Digital images were taken with light microscopy (Nikon Eclipse E600, SPOT Insight™ Camera, USA).

#### 2.4.2. Digestion of Hydrogels

The samples designated for biochemical assays were digested according to our previously established protocol [28], which was adopted and adjusted from a study in the literature [54]. Briefly, samples were dissolved and digested using a solution (pH = 7.5) containing 90 mM NaCl (Sigma-Aldrich), 100 mM sodium citrate (Sigma-Aldrich), 30 mM EDTA (Sigma-Aldrich), 0.1 mM Tris HCl (Sigma-Aldrich), 0.2 mM CaCl_2_ (Sigma-Aldrich), and 0.2 mg/mL proteinase K (Roche, Mannheim, Germany). Tubes containing the samples were primarily pipetted to dissolve the alginate hydrogel and then incubated at 55 °C overnight to further dissolve the hydrogel and digest the cells. As part of the experiments, PCL structures were removed from the tubes early in the dissolution process, weighed, and subtracted from the primary weight of the reinforced samples to get the weight of the hydrogel component only. The digested solutions were subjected to centrifugation at 10,000 rpm for 3 min, and dimethylmethylene blue (DMMB) and hydroxyproline (HP) assays were carried out separately on the digested solutions.

#### 2.4.3. Measurement of GAG Production

A DMMB assay was performed to measure GAG content within the constructs. We followed our previously established protocol [28], which was developed by combining and modifying several protocols [54,55,56,57]. Briefly, after transferring 50 µL aliquots of each digested solution to a 96-well plate, 200 µL of DMMB (Sigma, 341088) solution was added to each well and mixed by pipetting. Plates were incubated for 1 h at room temperature, and absorbance was measured by a microplate reader at 525 nm (Thermo Fisher). Based on the optical density (OD) values of the samples, concentrations of GAGs were quantified using a linear standard curve generated from various known concentrations of type A chondroitin sulfate (from the bovine trachea, Sigma-Aldrich). GAG content measured from digested solutions was normalized with hydrogel weights measured before the digestion step.

#### 2.4.4. Measurement of Total Collagen Production

Considering that HP is mostly located in collagen [58], HP content was used as an indicator of the total collagen content within the constructs. Our samples were analyzed using the assay, adhering to the protocol that was previously established [28]. HP was measured using a hydroxyproline assay kit (Abcam, ab222941). The remainder of the digested samples used for the DMMB assay were used for the HP assay. Digested samples were hydrolyzed using 10 N HCl at 100 °C for 4 h (1:1 ratio for sample volume to HCl volume ratio). Next, the samples were evaporated to dryness by heating at 65 °C for 2 h and were then oxidized by 100 μL of chloramine-T buffer (6 μL of chloramine-T concentrate mixed in 94 μL of oxidation buffer) at room temperature for 5 min. Then, 100 μL of dimethylaminobenzaldehyde (DMAB) solution (DMAB concentrate mixed in a developer solution (1:1)) was added and incubated for 90 min at 60 °C. Absorbance was measured at 560 nm in endpoint mode using a microplate reader. The HP concentrations of the solutions were determined using a linear standard curve created using known standard HP solutions with concentrations ranging from 0 to 50 µg/mL. A conversion factor of 7.6 was used to convert the HP content to the total collagen content of the solutions [59]. In the same way that GAG content was normalized, total collagen content measured from digested solutions was normalized by hydrogel weights measured before digestion.

#### 2.4.5. Evaluation and Quantitation of Immunofluorescence Staining

Depositions of Col1 and Col2 were detected by carrying out immunofluorescence staining on the sections according to the protocol in our previous study [28]. Briefly, the sections were digested with 0.1% trypsin (MP Biomedicals, 153571, Irvine, CA, USA), followed by treatment with 0.5% hyaluronidase (Worthington, Lakewood, NJ, USA). After blocking the sections with a solution of 4% natural goat serum (Sigma, G9023) and 2% natural sheep serum (Sigma, S3772) in 0.5% Triton X-100 in PBS (PBST), the sections were incubated with a solution containing primary antibodies of Col1 antibody (1:100, BioRad, 2150-1410, Hercules, CA, USA) and II-II6B Col2 antibody (1:100, DSHB, II-II6B, Iowa City, Iowa, USA) overnight at 4 °C. By doing so, both Col1 and Col2 were double immunostained on the same section. After additional washing steps with PBST, the sections were incubated with the blocking solution containing the secondary antibodies of goat anti-rabbit IgG-594 (1:1000, Invitrogen, applicable for primary anti-Col1) and goat anti-mouse IgG-488 (1:1000, Invitrogen, applicable for primary anti-Col2) for 3 h, in the dark, and at room temperature. DAPI (Vectashield, Vector Laboratories, Burlingame, CA, USA) was added to the stained sections, and fluorescence microscopy (Nikon, Eclipse E600, SPOT Insight™ Camera, USA) was used to take the images. Three images with different colors of red, green, and blue corresponded to Col1, Col2, and DAPI, respectively.

To quantify the immunofluorescence staining results, Adobe Photoshop software (Adobe Systems Inc., version 13.0, San Jose, CA, USA) was employed. Blue pixels on DAPI images were measured as representing the cell numbers on each image. To determine how much Col2 was deposited from the cell, the green colors in the Col2 images were quantitated, and the Col2/DAPI ratio was calculated. In Adobe Photoshop, pixels for positive red Col1 within the Col2 positive regions were measured, and % Col1/Col2 was calculated as a measure of produced fibrocartilage-like ECM to produce total cartilage-like ECM. It is also possible to calculate the amount of produced hyaline-like cartilage ECM by excluding the positive Col1 area from the positive Col2 area (% (1 − Col1/Col2)).

### 2.5. Terminology

Using the terminology introduced in the above section, cell numbers, produced fibrocartilage-like ECM, and produced hyaline-like cartilage ECM terms were used to refer to quantitative measurements for DAPI pixels, %Col1/Col2, and % (1 − Col1/Col2), respectively. The hydrogel constructs cultured for 5 and 10 days were referred to as unreinforced-5 days and unreinforced-10 days constructs, respectively, in the manuscript. Furthermore, reinforced hydrogel constructs cultured for 10 days were referred to as reinforced-10 days constructs.

### 2.6. Statistical Analysis

GraphPad Prism software package version 9.4.1 was utilized for performing statistical analyses. Given the small sample sizes across all groups, non-parametric statistical analysis using Kruskal-Wallis one-way analysis of variance (ANOVA) followed by Dunn’s multiple comparisons test was employed. The mean value with the corresponding standard deviation (SD) is presented for quantitative results within the context, while the figures depict the median and the interquartile range (IQR). Statistical significance was considered for *p*-values less than 0.05.

## 3. Results

### 3.1. GAG Deposition Confirmed Chondrogenic Differentiation

**Figure 2:**
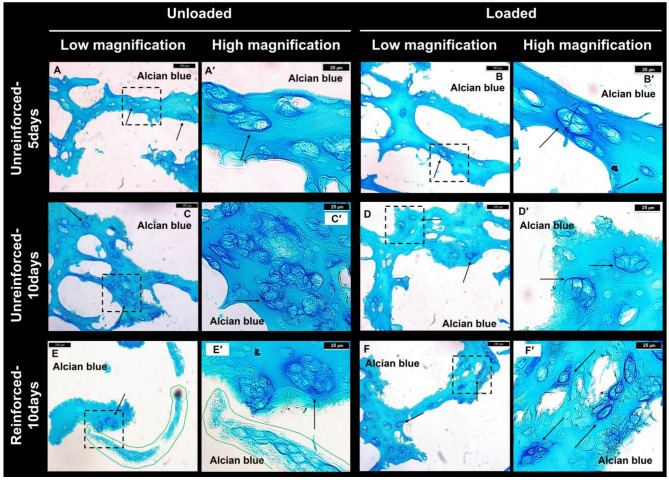
(**A**–**F**) Within different groups, Alcian blue-stained sections revealed GAG deposition occurring at various regions throughout the sections. (**A’**–**F’**) At higher magnification, the dashed rectangles within the images of (**A**–**F**) reveal GAG deposition surrounding the cells, characterized by a dark blue color. The arrows indicate the cells that have deposited GAGs. (Scale bars: (**A**–**F**) = 100 µm, and higher magnification images of (**A’**–**F’**) = 25 µm).

Sections generated from different construct groups were stained with Alcian blue to observe GAG deposition for ATDC5 cell’s chondrogenic differentiation. The alginate matrix was stained blue in the background, as was observed in our previous study as well. However, there were darker blue areas surrounding either single or clusters of cells, which distinguished the deposited GAG from the lighter blue background, indicating the differentiation of the cells to chondrogenic cells (Figure 2A–F,A’–F’, pointed to with arrows). Several clusters of cells were indeed observed at various regions of the constructs from all experimental groups. These clusters were dispersed throughout the hydrogel sections, and their formation could be attributed to either cell proliferation during the culture period or the initial impregnation process. As we attempted to ensure thorough cell dispersion before and during the mixing with the alginate solution, the abundance of these clusters at various regions suggests that cell proliferation is the primary factor contributing to their formation. (Figure 2A–F). The cell numbers were still low, as there were large areas of hydrogel matrix between cell clusters. It seemed that the cell numbers were lower in the unloaded and loaded un-reinforced-5 days constructs (Figure 2A,B) than the unreinforced-10 days and rein-forced-10 days constructs both in the unloaded and loaded conditions (Figure 2C–F). Elongated clusters of cells were observed in the sections from the reinforced constructs, which might have been attached to the PCL strands (Figure 2E,E’, solid red line).

### 3.2. GAG and Collagen Productions Tended Higher in Reinforced Constructs

**Figure 3:**
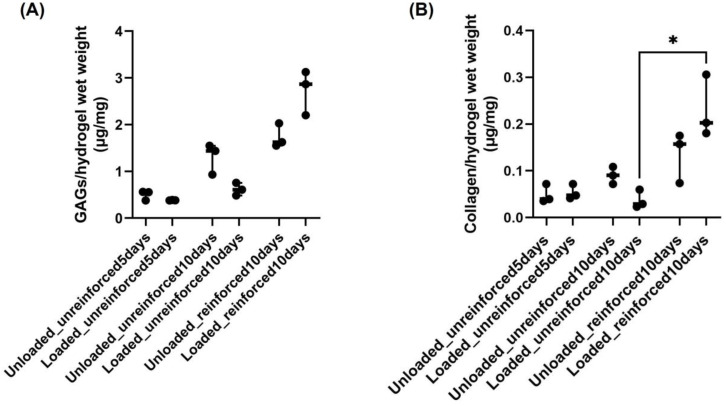
(**A**) Increasing the culturing period and using reinforced constructs trended to increase GAG production in constructs. (**B**) Collagen production was also enhanced by using reinforced constructs. Each dot represents different measurements for each experimental group (n = 3 for each group, * *p*  <  0.05).

As an assessment of chondrogenic differentiation cartilage ECM production, a DMMB assay was carried out to quantitate the levels of GAG contents within different constructs, unloaded and loaded. According to these results, loading conditions seemed to decrease GAG content in the unreinforced-5 days and -10 days constructs, while its content tended to increase in the loaded reinforced-10 days group compared to its unloaded group (Figure 3A). Extending the culturing period seemed to increase GAG contents in the unloaded and loaded unreinforced-10 days constructs compared to the unloaded and loaded, respectively, unreinforced-5 days constructs (Figure 3A). A similar trend was also seen by reinforcing the hydrogel constructs, as more GAG contents seemed to be present within the unloaded and loaded reinforced-10 days constructs compared to the unloaded and loaded, respectively, unreinforced-10 days constructs (Figure 3A).

By measuring the HP content of the constructs, the total collagen content within the constructs was indirectly measured, which was another important indicator of cartilaginous ECM. It seemed that collagen content did not differ by compression in the unreinforced-5 days constructs, but it tended to decrease in the loaded unreinforced-10 days constructs compared to the unloaded group (Figure 3B). Total collagen content seemed to increase within the loaded reinforced-10 days constructs compared to its unloaded group (Figure 3B). Extending the culture period seemed to increase the collagen content in the unloaded unreinforced-10 days constructs, but its content tended to decrease in the loaded unreinforced-10 days constructs compared to the loaded unreinforced-5 days constructs (Figure 3B). Reinforcing the hydrogel also seemed to enhance the collagen content of the reinforced-10 days constructs, as its content seemed to increase in the unloaded and loaded reinforced-10 days constructs compared to the unloaded and loaded, respectively, unreinforced-10 days constructs (Figure 3B). Although none of these observed changes in collagen content were statistically significant, there was a statistically significant increase in collagen content in the loaded reinforced-10 days constructs compared to the loaded unreinforced-10 days constructs (Figure 3B).

### 3.3. Reinforced Constructs Tending to Increase Cell Numbers and Fibrocartilage Formation

**Figure 4:**
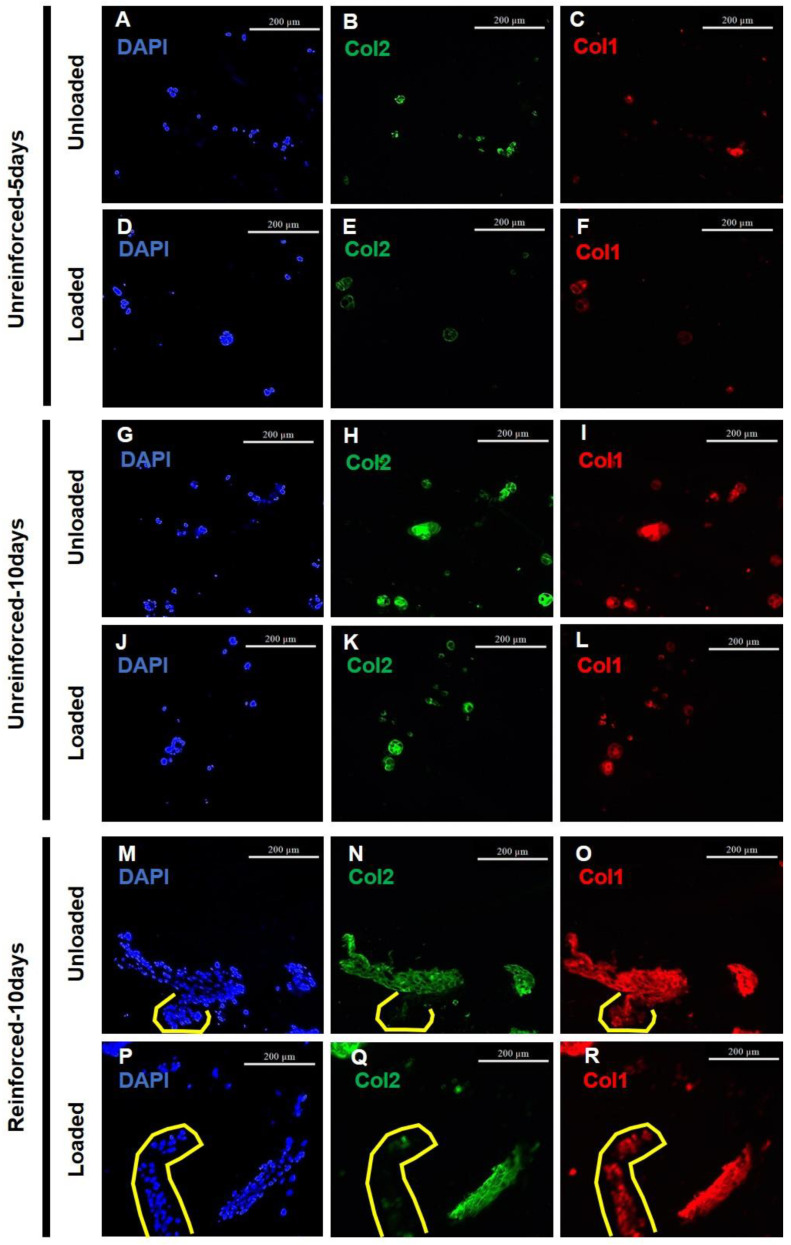
(**A**,**D**,**G**,**J**,**M**,**P**) Immunofluorescence staining using DAPI demonstrated that the cell numbers appeared to be more abundant in the reinforced constructs compared to the other groups. (**B**,**E**,**H**,**K**,**N**,**Q**) Col2 immunofluorescence staining was positive for most of the cells in the un-reinforced groups, whereas some cells in the reinforced ones did not deposit Col2. (**C**,**F**,**I**,**L**,**O**,**R**) Col1 immunofluorescence staining revealed the deposition of Col1 by a significant number of cells in all groups, indicating the differentiation of cells into fibrochondrocytes. (Scale bars = 200 µm).

The deposition of Col1 and Col2 by the impregnated cells was visualized through immunofluorescence staining of sections in different groups (Figure 4). Most of the ATDC5 cells showing Col2 deposition also stained positively for Col1, indicating they were predominantly differentiated to fibrochondrocytes (Figure 4). According to the DAPI images, both loaded and unloaded reinforced-10 days groups showed high cell numbers (Figure 4). Sections from the loaded and unloaded reinforced-10 days groups also showed more aggregated cells (Figure 4). The depositions of Col1 in unreinforced-5 days and -10 days groups were associated with the deposition of Col2 (Figure 4), whereas in reinforced groups, some cells deposited Col1 without depositing any Col2 (Figure 4M–R, indicated by yellow lines). Col1 deposition also appeared to be stronger in unloaded and loaded reinforced-10 days groups (Figure 4) than in unloaded and loaded unreinforced-5 days and -10 days groups.

**Figure 5:**
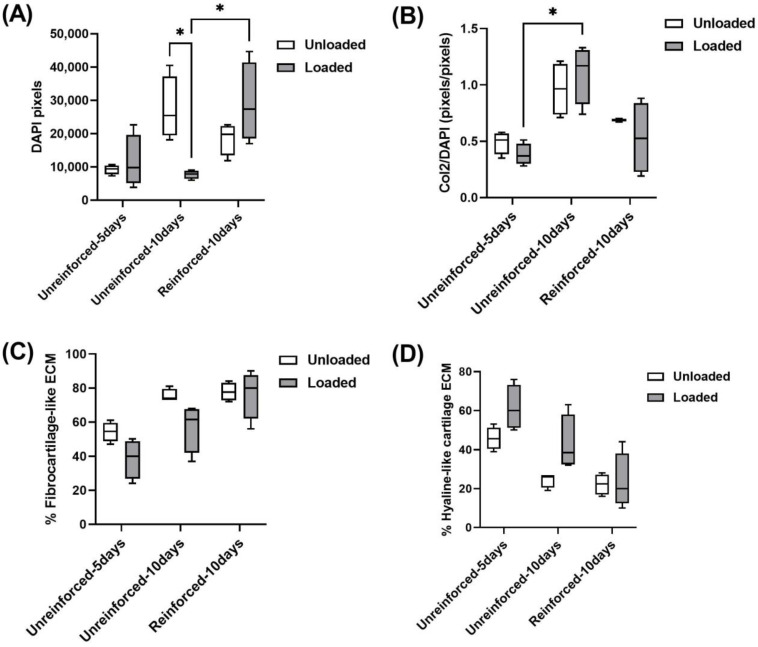
(**A**) Quantitative results showed that lengthening the culture period seemed to increase Col2 deposition in unreinforced constructs, and reinforced constructs trended to produce lower Col2 than the unreinforced constructs. (**B**) Cell numbers decreased by loading condition in the 10 days unreinforced group, whereas they increased in the loaded reinforced group. (**C**) Fibrocartilage-like ECM production tended to increase by increasing the loading period and reinforcing the constructs. (**D**) Hyaline-like cartilage ECM production was highest in 5 days of unreinforced constructs and lowest in reinforced groups. (n = 4 for each group, and * *p* < 0.05).

Using Adobe Photoshop software, immunofluorescence images were quantitated to determine how cell numbers and ECM production changed. Based on DAPI pixel quantitation, loading conditions seemed to slightly increase cells in the unreinforced-5 days constructs without any statistical significance (Figure 5A). In contrast, the loaded unreinforced-10 days constructs showed a statistically significant decrease in cell numbers compared to the unloaded unreinforced-10 days constructs (Figure 5A). The cell population in the reinforced-10 days constructs appeared to be higher compared to their unloaded counterparts, but the difference was not statistically significant (Figure 5A). Extending the culture period appeared to have a positive effect on the cell numbers in the unloaded unreinforced-10 days constructs compared to the unloaded unreinforced-5 days constructs. However, it seemed to have a negative effect on the cell numbers in the loaded unreinforced-10 days constructs compared to the loaded unreinforced-5 days constructs. It’s worth noting that none of these changes showed statistical significance (Figure 5A). Reinforcing appeared to result in a decrease in cell numbers when compared to the unloaded, unreinforced-10 days constructs, but this change was not statistically significant. However, in the loaded reinforced-10 days constructs, reinforcement led to a statistically significant increase in cell numbers compared to the loaded unreinforced-10 days constructs (Figure 5A).

Col2 deposition normalized to DAPI staining (Col2/DAPI) tended to decrease in the loaded unreinforced-5 days constructs compared to their unloaded group (Figure 5B). In contrast, Col2/DAPI seemed to increase with loading conditions in the unreinforced-10 days constructs (Figure 5B). In the reinforced constructs, the loading conditions seemed to decrease Col2/DAPI deposition, but these observed changes were not statistically significant within any of the groups due to loading (Figure 5B). Col2/DAPI tended to increase by extending the culturing period in both unloaded and loaded unreinforced-10 days constructs compared to the unloaded and loaded, respectively, unreinforced-5 days groups (Figure 5B). Furthermore, the increase in Col2/DAPI deposition within the loaded unreinforced-10 days constructs compared to the loaded unreinforced-5 days group was statistically significant. On the other hand, reinforcing the hydrogels appeared to cause a reduction in Col2/DAPI levels in both the unloaded and loaded reinforced-10 days constructs when compared to their respective unreinforced-10 days constructs. However, these changes were not statistically significant (Figure 5B).

The areas where Col2 staining was positive and Col1 staining overlapped (Col1-positive pixels within Col2-positive pixels) were identified as regions containing fibrocartilage-like ECM. Quantitative analysis was then performed on these areas. Furthermore, quantitation was done for areas where Col2 was positive but Col1 was negative (Col1-negative but Col2-positive pixels), which produced hyaline-like cartilage ECM. The evaluation of fibrocartilage-like ECM and hyaline-like cartilage is crucial for understanding the cartilage regeneration process. A decrease in fibrocartilage-like ECM production is desirable as it indicates a shift towards the production of hyaline-like cartilage, which is crucial for cartilage regeneration. Conversely, an increase in fibrocartilage-like ECM production is not favorable for the purpose of hyaline cartilage regeneration. Therefore, it is essential to analyze and interpret the changes in ECM production accurately. Quantitation for fibrocartilage-like ECM production showed that it seemed to decrease in the loaded unreinforced-5 days constructs compared to their unloaded group (Figure 5C). Similar to the unreinforced-5 days group, loading conditions tended to decrease fibrocartilage-like ECM production in the loaded unreinforced-10 days constructs compared to their unloaded group (Figure 5C). In the reinforced-10 days constructs, loading conditions appeared to cause a minor reduction in fibrocartilage-like ECM production from 0.78 ± 0.05 under unloaded conditions to 0.76 ± 0.14 under loaded conditions (Figure 5C). Extending the culture period seemed to increase fibrocartilage-like ECM production in both unloaded and loaded unreinforced-10 days constructs compared to the unloaded and loaded, respectively, unreinforced-5 days constructs (Figure 5C). Reinforcing the constructs also seemed to increase fibrocartilage-like ECM production in both unloaded and loaded reinforced-10 days constructs compared to the unloaded and loaded, respectively, unreinforced-10 days constructs, although the increase in the unloaded reinforced-10 days group compared to the unloaded unreinforced-10 days group was very slight (Figure 5C). It should be noted that the observed changes were not statistically significant.

The quantitation of hyaline-like cartilage ECM production showed an inverse trend compared to the percentage of fibrocartilage-like ECM, as these measurements represented complementary percentages. Loading conditions seemed to increase hyaline-like cartilage ECM production within all groups, although the increase was slight in the loaded reinforced-10 days group compared to its unloaded group (Figure 5D). Extending the culture period and reinforcing the hydrogels both seemed to reduce hyaline-like cartilage ECM production within the respective unloaded and loaded constructs of the unreinforced-10 days and reinforced-10 days groups. The changes observed were not statistically significant (Figure 5D).

## 4. Discussion

CTE researchers have been increasingly interested in using hydrogels since they provide an environment that is highly hydrated for cells to reside in [1]. For CTE, the general approach is to impregnate the cells into hydrogels for hyaline cartilage regeneration. Fabricated hydrogel constructs are supposed to be implanted in the joint where mechanical forces are applied to them. It is worth noting that hydrogels lack the mechanical properties necessary to support load-bearing capacity, which means they may lose their 3D integrity in vivo long before sufficient cartilage would have been regenerated to sustain them [60]. Additionally, impregnated cells are susceptible to forming Col1, which leads to fibrocartilage ECM production, when high mechanical forces are applied to the hydrogels [45,46,47].

Our study aimed to use a bioreactor system to observe cartilage ECM production in unreinforced and PCL-reinforced hydrogel constructs in response to applied compressive forces. Histological staining with Alcian blue confirmed chondrogenic differentiation of the impregnated ATDC5 cell lines. GAG deposition was evident across all groups, confirming that cell differentiation occurred in both the unloaded and loaded conditions of both unreinforced and reinforced constructs. Unreinforced-10 days constructs showed more clusters of GAG-depositing cells, suggesting that extending the culture period could increase cell numbers and facilitate differentiation. There were elongated clusters of cells that appeared to be attached to PCL strands as if the cells migrated toward the strands and attached to them. However, the reason for this phenomenon remains unknown.

The biochemical evaluation using DMMB and HP assays confirmed the production of GAGs and collagens, which are essential components of the cartilage ECM. Although loading seemed to increase GAG and collagen production within the reinforced-10 days constructs, it seemed that loading regimes either reduced or did not alter the GAG and collagen content of the loaded unreinforced-5 days and -10 days constructs. It has been reported that cyclic compression greatly increases the release of cells and newly synthesized ECM molecules from hydrogels [61]. The loss of cells and ECM macromolecules may result from both the movement of fluids through the interconnected pores and networks within the hydrogels and from the mechanical disruption of newly synthesized ECM [62,63]. Thus, mechanically loaded 3D-bioprinted hydrogels might lose their 3D integrity and degrade due to compression forces leading to ECM release from their structure. Furthermore, extended compression might facilitate this process, as a higher reduction was seen in the loaded unreinforced-10 days constructs as compared to their corresponding unloaded group, while the reduction was slighter in the loaded unreinforced-5 days constructs. In contrast, reinforcing seemed to help in retaining higher GAG and collagen content, as both unloaded and loaded reinforced-10 days constructs contained higher GAG and collagen content compared to the unloaded and loaded, respectively, unreinforced-10 days constructs. PCL-reinforced-10 days constructs lack large pores as do 3D-bioprinted unreinforced-10 days hydrogels, and hence reinforced-10 days constructs exhibit less degradability and may retain synthesized ECM more efficiently.

Immunofluorescence stained images were analyzed visually and quantitatively to find out how the cell numbers changed and how much hyaline-like cartilage vs. fibrocartilage-like ECM was produced. DAPI staining was performed and quantitated, as higher cell numbers are necessary for cartilage matrix formation and in vivo cartilage regeneration. It seemed that the loading conditions did not alter the cell numbers very much in the unreinforced-5 days constructs and decreased them in the loaded unreinforced-10 days constructs, whereas cell numbers seemed to increase by loading the reinforced-10 days constructs. Extended loading conditions might not cause cell death in the loaded unreinforced-10 days constructs, as it was reported in the literature that dynamic strain increases DNA synthesis in cells and boosts their proliferation in hydrogels rather than causing cell death [62,64]. The unreinforced-10 days constructs might lose their 3D integrity and degrade more in response to an extended loading period, which in turn would lead to cell migration out of the hydrogel structure. Furthermore, attached cells were observed on the surface of 6-well plates of the loaded unreinforced-10 days constructs, which indicated cells out of the constructs. In contrast, the loaded, reinforced-10 days constructs have higher mechanical properties and do not lose their hydrogel compartment while being compressed, which helps in retaining the proliferated cells. Furthermore, attached cells were not observed within the 6-well plates of the loaded, reinforced-10 days constructs. Maintaining sufficient numbers of cells within the constructs is crucial for in vivo implantations and promoting the formation of cartilage ECM [6]. Loaded reinforced constructs are preferable in this regard because they show the highest cell numbers between the loaded constructs. Although extending the culture period seemed to increase cell numbers in the unloaded unreinforced-10 days constructs as compared to the unloaded unreinforced-5 days constructs, it seemed that extending is not beneficial for loaded constructs since it causes cell migration from them. Conversely, reinforcing seemed beneficial for loaded conditions, but it decreased cell numbers in the unloaded condition of the reinforced-10 days constructs compared to the unloaded unreinforced-10 days constructs. Since the unloaded unreinforced-10 days and unloaded reinforced-10 days constructs were cultured in the same conditions, it can be concluded that the cells proliferate more within the unreinforced hydrogel constructs, whereas reinforcing inhibits cell growth within the reinforced constructs. This may be related to the higher stiffness of the reinforced construct, which could inhibit cell growth. It was also reported in the literature that cell proliferation is downregulated in hydrogels with a lower gel relaxation rate as compared to softer hydrogels [38]. Our reinforced-10 days constructs are also considered slower-relaxing hydrogel constructs as compared to the unreinforced-10 days constructs.

Col2 deposition was analyzed as an important indicator of the chondrogenic differentiation of the cells. Across all groups, most of the ATDC5 cells deposited Col2, which was further evidence that they were chondrogenically differentiated. The deposition of Col2 was observed to be localized around the cells, and it did not appear to diffuse into the alginate matrix. The utilization of the ATDC5 cell line in this particular investigation might have influenced a diminished production of cartilage ECM, as observed. Prior studies have indicated that the application of alternative cell sources, such as non-immortalized stem cells or primary cells, in hydrogel constructs could potentially enhance the regeneration of the cartilage matrix [65,66]. Loading did not result in any statistically significant differences in the quantitation of Col2/DAPI, although minor non-significant differences were observed between the loaded and unloaded constructs of different groups. While most studies reported upregulation of Col2 deposition in loaded hydrogels [67,68,69], some studies found similar results to what is reported here [70,71] as Col2 deposition seemed to slightly decrease in the loaded unreinforced-5 days and loaded reinforced-10 days constructs. Culturing factors such as cell type, hydrogel properties, and dynamic culturing conditions, which may differ across experiments, influence the mechanotransduction pathways of the cells and alter Col2 production. Hence, it seems necessary to examine pathways that regulate Col1 and Col2 production within these constructs, both loaded and unloaded. Extending the loading period enhanced Col2/DAPI production within the unreinforced constructs, which is a positive outcome for cartilage regeneration. The reason might be that ATDC5 cells were sufficiently proliferated to initiate chondrogenesis by static pre-culturing, and their differentiation was enhanced by further dynamic culturing [72]. Enhanced chondrogenesis by extending the compression periods was also reported in earlier studies [73]. Furthermore, hydrogel degradation due to the extended culture period would decrease the mechanical properties of the unreinforced-10 days constructs, leading to changing mechanical cues sensed by the cells and more Col2 depositions. It is important to note that the Col2/DAPI ratio represents a normalized quantity, indicating the amount of Col2 production per cell. Therefore, even though the cell numbers decreased in the loaded unreinforced-10 days constructs, potentially due to cells being pushed out of the constructs, the remaining cells inside the constructs exhibited an enhanced capacity for Col2 deposition in response to the mechanical forces applied. On the other hand, the reinforced-10 days constructs seemed to deposit less Col2 than the unreinforced-10 days constructs, both in the unloaded and loaded conditions. This finding suggests that the presence of reinforcement, in the form of PCL, may have a negative impact on the production of cartilage matrix. This may relate to the higher mechanical properties of the reinforced constructs, such as higher stiffness and lower stress relaxation. It has been reported that both cell proliferation and Col2 deposition are greater in structures that relax rapidly, such as softer hydrogels than in those that relax slowly [38]. However, we must note that the exact mechanisms underlying this observation require further investigation. The interplay between mechanical properties and chondrogenic outcomes is complex, and additional studies are needed to fully understand the underlying factors influencing Col2 deposition. Furthermore, exploring the mechanical properties of both reinforced and unreinforced constructs at different time points, along with assessing cellular behavior and ECM production, could be a valuable avenue of research. This suggestion opens possibilities for future studies to delve deeper into understanding how mechanical properties influence cellular response and tissue development over time.

The amount of Col1 deposited from the cells leading to fibrocartilage-like ECM production was also examined by immunofluorescence staining. Most of the cells seemed to differentiate into fibrochondrocytes by depositing both Col1 and Col2. Quantitation of fibrocartilage-like ECM showed that loading conditions seemed to decrease its production in unreinforced-5 days and -10 days constructs, whereas loading seemed not to alter fibrocartilage-like ECM production in the loaded unreinforced-10 days constructs. These findings suggest that compression, applied through loading conditions, is beneficial for reducing fibrocartilage-like ECM production in the unreinforced constructs. However, it appears that the reinforced constructs may not respond as favorably to the applied compression in terms of reducing fibrocartilage-like ECM production. Previous studies also reported downregulation or no changes in Col1 production in response to the mechanical loading of hydrogel constructs [67,74]. The deposition of Col1 in reinforced constructs was not only stronger in immunostained images, but cells in multiple regions appeared to deposit Col1 without any Col2 deposition, which was not common in the unreinforced constructs. It also seemed that reinforced constructs produced a higher amount of fibrocartilage-like ECM among all groups. This could be related to the mechanical properties and stiffness of the reinforced constructs. Previously, the stiffness of constructs was shown to influence cartilage formation in vitro [75,76]. Arora et al. and Toh et al. also reported that stiff constructs exhibited the highest Col1 deposition [76,77]. Extending the culture period and reinforcing the constructs both seemed not to help in reducing fibrocartilage-ECM production. In order to decrease the production of fibrocartilage-like ECM, further improvements are required, whether through changes in the type of impregnated cells or the constructs used.

**Figure 6:**
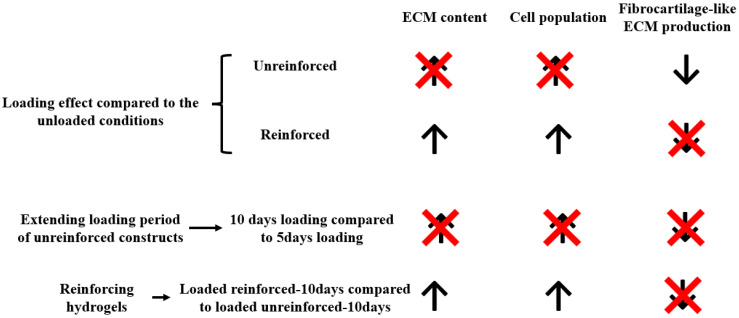
Summary of the main findings. Unreinforced constructs seemed to reduce fibrocartilage-like ECM production when they were loaded but did not seem to increase cell numbers or ECM contents. Conversely, the reinforced constructs seemed to be beneficial for mechanically loaded conditions, including in vivo implantations, since they seemed to increase cell numbers and ECM content, but fibrocartilage-like ECM production is high. Upward arrows represent an increase in cell numbers and ECM content, while upward arrows with ‘×’ indicate no change or reduction. The downward arrow signifies a desired decrease in fibrocartilage-like ECM production, and downward arrows with ‘×’ represent no change or reduction.

In order to make the findings of this study more accessible to readers, a summary figure (Figure 6) has been included. This figure is based on the desired outcomes that the researchers hoped to see, such as an increase in cell numbers and ECM content, which are represented by upward arrows. Conversely, if there was a reduction or no change in these desired outcomes, they are presented as an upward line with a multiplication sign (‘×’). We also aimed for a decrease in fibrocartilage-like ECM production, which is indicated by a downward arrow in the figure. If there was no change or reduction in fibrocartilage-like ECM production, then it was shown by a downward sign with a multiplication sign. However, it should be noted that this figure is not based on statistical significance, as the sample numbers were small and statistical significance was difficult to obtain. Instead, the figure is based on the perceived results observed from the outcomes.

## 5. Conclusions

Hydrogel constructs are susceptible to losing cells and ECM while also producing fibrocartilage ECM in response to mechanical compression. The reinforcement of hydrogels with a 3D-printed structure may improve their performance, but changes in fibrocartilage formation are an open question to be explored. Thus, this study examined how the cell numbers and amount of the ECM produced as well as its type changed by first extending the culture period in 3D-bioprinted unreinforced hydrogel constructs and secondly using hydrogels reinforced within 3D-printed PCL structures in comparison to 3D-bioprinted hydrogels. Chondrogenic differentiation of the impregnated ATDC5 cells was confirmed by the production of GAGs and Col2 through histological staining, biochemical assessment, and immunofluorescence staining. It seemed that not only did extending the culture period increase ECM content, but using a reinforced construct also helped in this regard. The loaded, unreinforced-10 days constructs did not only have lower cell numbers but also released cells in their culture media. A higher number of cells, however, was measured within the loaded reinforced constructs, indicating that reinforced constructs were more capable of both stimulating cell growth and retaining the proliferated cells. Greater cell numbers and ECM content in the reinforced constructs are beneficial features for in vivo cartilage regeneration. Despite these advantages, reinforced constructs still did not seem to produce higher Col2 levels, while fibrocartilage-like ECM production seemed to be quite high. In contrast, the loaded unreinforced constructs, which have softer structures, produced more Col2 while producing lower amounts of Col1 and fibrocartilage-like ECM. Research into the mechanical properties of the reinforced constructs is recommended to adjust those properties for increasing Col2 and reducing Col1 production. In addition, finding the mechanotransduction pathways causing Col1 production in reinforced constructs and blocking them may help produce more hyaline-like cartilage ECM.

## Data Availability

The data that support the findings of this study are available upon reasonable request from the corresponding author.
